# Computer Vision over 4G/5G Private Network for Real-Time Draft Assessment to Estimate Vessel Productivity

**DOI:** 10.3390/s26082443

**Published:** 2026-04-16

**Authors:** Tiago Novaes Mathias, Hugo da Silva Bernardes Gonçalves, Vinícius Veiga Paschoal, Danillo Henrique Araujo de Lima, Júlio César de Oliveira Duque, Arthur Lotenberg, Willian Elias Araújo, Rui Carlos Botter, Daniel de Oliveira Mota

**Affiliations:** 1 Infrastructure Digital Transformation Engineering Department, Port and Airport Research Institute, Yokosuka 239-0826, Japan; 2 Applied Computing, National Institute for Space Research, São José dos Campos 12227-010, Brazil; hugo.bernardes@gmail.com; 3 Department of Electrical and Automation Engineering, University of São Paulo, São Paulo 05508-010, Brazil; vinicius.vp@usp.br; 4 Department of Production Engineering, University of São Paulo, São Paulo 05508-010, Brazil; danillohal@usp.br (D.H.A.d.L.); oficialjulioduque@usp.br (J.C.d.O.D.); arthur.lottenberg@usp.br (A.L.); danielmota@usp.br (D.d.O.M.); 5 NLT Next Level Telecom, São Paulo 04576-060, Brazil; william.araujo@nlt.com.br; 6 Department of Naval and Ocean Engineering, University of São Paulo, São Paulo 05508-010, Brazil; rcbotter@usp.br

**Keywords:** draft survey, automated draft survey, ship draft survey, vessel inspection, computer vision, hull segmentation, YOLOv8, port operations monitoring

## Abstract

Background: Draft surveys are widely used to estimate cargo mass during bulk vessel loading and unloading; however, conventional procedures depend on manual draft readings that are episodic, labor-intensive, and sensitive to environmental conditions. Existing camera-based automated approaches rely on draft mark recognition or explicit waterline detection, which remain vulnerable to illumination variability, hull fouling, and wave-induced disturbances. Methods: This paper proposes a computer vision framework deployed at the Port of Santos, Brazil, using fixed quay-side cameras and a private 4G network infrastructure for continuous image transmission. Unlike prior methods, the framework estimates emergent hull height by segmenting vessel hull contours from bow and stern viewpoints using customized YOLOv8 instance-segmentation models, without relying on draft marks or waterline detection. Pixel measurements are converted to metric units using a nearby bollard of known height as a local physical reference. Results: Field experiments monitor a Panamax bulk carrier over approximately 6.5 days, processing more than 34,000 images from each camera at an average rate of 3.7 images per minute. Both bow and stern segmentation models achieve mAP50-95 mask scores of 0.980 and 0.965, respectively, confirming precise and stable hull boundary delineation. Hull height decreases from 8.27 m to 4.64 m at the bow and from 7.98 m to 3.98 m at the stern over the loading period, with coherent and temporally stable trends across independent viewpoints. Conclusions: The proposed approach delivers repeatable and continuous hull-height estimates under real operational conditions, including variable lighting, background clutter, and partial occlusions, offering a practical and non-intrusive complement to traditional draft surveys for continuous vessel loading monitoring in modern ports.

## 1. Introduction

Accurate determination of cargo mass during bulk loading and unloading operations is a critical requirement for maritime logistics, commercial settlement, and regulatory compliance. The draft survey remains the most widely adopted method for estimating cargo weight in bulk shipping, relying on hydrostatic principles to infer vessel displacement from measured drafts before and after cargo operations. Despite its widespread acceptance, the traditional draft survey is labor-intensive, time-constrained, and highly sensitive to environmental conditions, surveyor subjectivity, and hull-related uncertainties such as trim, list, and hogging or sagging effects [1,2,3,4,5]. These limitations have motivated sustained research into automated and camera-based alternatives capable of improving objectivity, safety, and temporal resolution.

Recent industry guidance and empirical studies emphasize that draft reading itself constitutes the dominant source of uncertainty in draft surveys, particularly under adverse weather, poor visibility, nighttime operations, or when draft marks are obscured by fouling or hull paint degradation [2,6,7]. While auxiliary instruments such as manometers, laser levels, and drones have been introduced to mitigate some of these challenges, they remain largely episodic tools rather than continuous monitoring solutions. At the same time, the increasing operational tempo of modern ports and the growing relevance of digitalization in maritime infrastructure demand measurement systems that operate continuously, non-intrusively, and with minimal human intervention.

Advances in computer vision and deep learning have significantly expanded the feasibility of automated draft observation. Early image-processing approaches emulated manual draft reading through morphological filtering, edge detection, and optical character recognition, achieving centimeter-level accuracy under controlled conditions [8,9,10]. Li et al. [11] demonstrated the inherent complexity of modeling water surfaces from a single viewpoint, highlighting challenges such as wave dynamics and specular reflections that directly undermine the reliability of waterline-dependent draft measurement approaches. More recent systems integrate convolutional neural networks for digit recognition, waterline segmentation, and end-to-end draft estimation, demonstrating millimeter-scale precision in favorable environments [12,13,14,15]. However, most existing methods continue to depend on explicit detection of draft marks and the waterline, which remain vulnerable to illumination variability, wave-induced reflections, and hull surface degradation. Moreover, these systems are typically evaluated at discrete survey moments rather than as part of a continuous automated operational monitoring framework. Some research has demonstrated the potential of continuous video-based water surface tracking in controlled maritime experimental environments [16], further motivating the development of operational monitoring systems capable of sustained deployment in real port conditions.

In parallel, the YOLO (You Only Look Once) framework family of real-time object detection and segmentation models has emerged as a practical foundation for deployable vision systems, balancing inference speed, accuracy, and architectural simplicity [17,18,19,20,21,22]. Instance segmentation variants, in particular, enable pixel-level delineation of objects and open new possibilities for geometric measurement beyond traditional bounding-box-based detection. This capability is especially relevant in maritime environments, where stable structural elements of vessels may be more reliably segmented than fine-scale markings.

Considering this background, the following research question motivates this work: Can a computer vision framework based solely on hull contour segmentation, without relying on draft mark recognition or explicit waterline detection, provide reliable and continuous estimates of vessel loading state under real operational port conditions?

To address this question, this paper proposes a novel framework for automated hull height estimation that combines fixed quay-side cameras, a private 4G communication network, and customized YOLOv8 instance-segmentation models. Rather than detecting draft marks or waterlines, the method estimates the emergent hull height by segmenting the visible hull contour and converting pixel measurements into metric units through a locally available fixed object of known height as a physical reference.

Furthermore, the proposed framework integrates three key components: (i) a secure and resilient data transmission architecture based on a private 4G network; (ii) viewpoint-specific customized hull segmentation using deep learning; and (iii) a geometric measurement pipeline that extracts repeatable hull-height estimates from static camera images. Field experiments conducted at the Port of Santos demonstrate the system’s ability to operate continuously over multiple days, producing temporally consistent hull measurements from independent bow and stern viewpoints. While not intended to replace statutory draft surveys at this stage, the proposed system provides a foundation for continuous monitoring of vessel loading states and offers a complementary digital instrument for port authorities, terminal operators, and surveyors.

The main contributions of this work are summarized as follows:

- A mark-free hull-height estimation method: A geometric measurement pipeline that estimates emergent hull height directly from segmented hull contours, requiring no draft mark detection, digit recognition, or explicit waterline identification, removing the most uncertainty-prone step in traditional and existing automated draft survey approaches.
- A private 4G network architecture for continuous port monitoring: A secure and resilient data transmission system enabling uninterrupted image acquisition and live transfer in a real operational port environment, without reliance on public cellular infrastructure.
- Viewpoint-specific YOLOv8 segmentation models: Customized instance-segmentation models trained independently for bow and stern viewpoints, achieving mAP50-95 mask scores of 0.980 and 0.965, respectively.
- Field validation at the Port of Santos: An end-to-end experimental demonstration on a bulk carrier, producing consistent hull-height time series from independent bow and stern viewpoints under real conditions including variable lighting, background clutter, and partial occlusions.

To improve readability, this paper is organized as follows. After the introduction in Section 1, Section 2 provides a brief literature review and defines the main concepts regarding draft survey and computer vision. Section 3 then explains the proposed methodology. Thereafter, Section 4 describes the experimental setup, followed by Section 5 with the preliminary results. Subsequently, Section 6 and Section 7 present the discussion and conclusions.

## 2. Literature Review

### 2.1. General Principles of Draft Survey Calculations

A draft survey determines the mass of cargo loaded or discharged by comparing the vessel’s displacement before and after cargo operations, applying Archimedes’ principle: a floating body displaces a volume of water whose weight equals the total weight of the ship and everything on board. The surveyor, therefore, (i) measures the ship’s drafts, (ii) uses hydrostatic data to obtain the corresponding displacement, and (iii) deducts the weights of ballast, bunkers, fresh water, stores, and crew (“deductibles”) to isolate the cargo weight [4]. Standard practical procedures [2] emphasize careful preparation, stable loading conditions, and strict adherence to trim and list limits to achieve the accuracy required for commercial bulk trades.

In practice, a draft survey begins with reading the draft marks at six standard locations: forward, midships, and aft, on both the port and starboard sides. These six readings allow the surveyor to determine both the vessel’s overall trim and any longitudinal hull deflection. The hull may be hogged, curved upward in the midship region relative to the ends, or sagged, curved downward in the middle so that the midship draft is greater than expected from the end drafts. Ref. [2] formalizes this using the Arithmetical Mean Draft (AMD), defined as the mean of the corrected forward and aft drafts: if the actual midship draft is less than AMD, the ship is hogged; if it is greater, the ship is sagged. Hogging and sagging arise from non-uniform distribution of cargo, ballast, and bunkers; residual structural deformation; and diurnal thermal effects on large hulls. These deformations affect immersed volume and must be corrected through “mean-of-means” or equivalent hull-deflection correction methods before entering hydrostatic tables [1].

**Figure 1:**
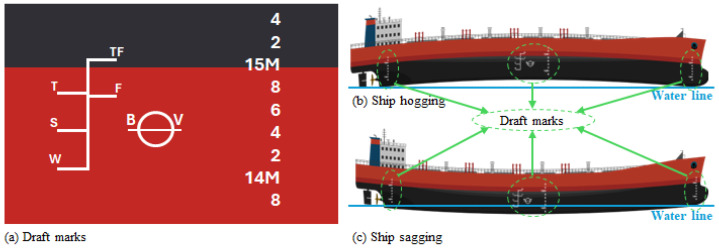
Example of draft mark locations and hull-deflection conditions.

Figure 1a shows a schematic representation of a bulk carrier showing the six standard draft-reading positions (forward-port, forward-starboard, midship-port, midship-starboard, aft-port, aft-starboard). The Figure 1b inset illustrates hogging and Figure 1c shows sagging conditions, with relative midship draft deviations referenced to the AMD. Such diagrams help visualize the geometric implications of hull curvature for displacement determination.

The environmental conditions at the time of reading the drafts are a source of uncertainty. Guidelines and empirical studies highlight the adverse influence of swell, short-period waves, tidal currents, wind loading, poor visibility, rain, snow, and fouled or poorly painted marks. Waves cause the waterline to oscillate relative to the marks; currents and wind induce dynamic trim and heel; strong sunlight or darkness obscure graduations; and marine growth or rust may cover numbers. Ref. [2] recommends reading drafts at slack water, when possible, using boats or ladders to view offshore marks directly, and documenting sea and weather conditions in the survey report. Modern practice also uses equipment such as manometers for outboard drafts, calibrated hydrometers for water density, and increasingly high-zoom cameras and drones for capturing clear, repeatable images of draft marks.

Protection and Indemnity (P&I) clubs play a central role in the legal environment surrounding draft surveys, covering shipowners’ third-party liabilities, including cargo-shortage claims resulting from discrepancies between shore-scale and draft-survey weights. Standardized procedures, environmental documentation, and clear records are, therefore, essential to reduce errors and mitigate commercial disputes. According to Japan P&I, approximately 21% of cargo-related claims in a multiyear study involved alleged shortages, resulting in compensation payments totaling tens of millions of dollars [7]. Accordingly, Britannia Loss Prevention provides detailed guidance documents, emphasizing standardized procedures, environmental documentation, and clear records to reduce errors and mitigate disputes [2,6,23].

Research into error propagation in draft surveys identifies four principal sources: draft reading; ballast-tank measurement; water-density determination; and hydrostatic-table interpolation [7]. Among these, draft reading has been consistently identified as the most critical stage, with surveyor competence, difficulty reading seaward marks, and adverse weather conditions being the most influential sub-criteria. Small inaccuracies of a few centimeters in draft readings can dominate total displacement error, particularly when combined with hull deflection or trim [3,24].

Such small inaccuracies of a few centimeters can propagate into displacement and cargo-weight errors that dominate total displacement uncertainty, especially when combined with hull deflection or trim [24]. Researchers have proposed a fiber-optic draft-reading system to reduce subjectivity and improve repeatability. Wawrzyński [3] similarly demonstrates that neglecting second-trim corrections leads to systematic bias, particularly in stern-trimmed vessels. Liu et al. [25] show that densifying offset tables and generating high-resolution hydrostatic datasets reduces hydrostatic-interpolation errors to about 0.01% for underwater volume, waterline area, and TPC, addressing one of the last persistent systematic error sources in traditional draft surveys.

Finally, both industry practice and the recent literature indicate growing integration of enhanced measurement tools and partial automation within traditional draft surveys. Dibble [2] notes the use of manometers, float-type devices, and photographic documentation to mitigate parallax and wave impacts. Practical guidance from Britannia [6] and Ruswal [26] shows high-zoom cameras, drones, and laser levels, especially at night or where draft marks are inaccessible. These developments form a natural bridge toward fully automated draft-survey systems based on digital imaging and computer vision, which will be examined in the next subsection [4].

### 2.2. Brief Overview of You Only Look Once—YOLO

The You Only Look Once (YOLO) family represents a major line of research in real-time object detection by framing detection as a single-stage learning problem. In contrast to two-stage paradigms, for example, region proposal followed by classification, the original YOLO formulation casts detection as direct regression from an input image to bounding box coordinates and class probabilities in one forward pass, enabling end-to-end optimization and high throughput suitable for time-sensitive applications. This “single-shot” design has helped establish YOLO as a practical baseline for operational computer vision systems where latency, hardware constraints, and deployability are as important as accuracy [17,18,19].

A core progression in YOLO concerns how candidate objects are parameterized and predicted across scales. YOLOv2/YOLO9000 introduced widely adopted improvements that supported stronger accuracy–speed tradeoffs and expanded detection vocabulary by combining detection and classification supervision [18]. Further, YOLOv3 consolidated YOLO as a competitive real-time detector by adopting anchor-based bounding box prediction using priors and logistic activations, multi-label class prediction via independent logistic classifiers (rather than softmax), and multi-scale prediction to improve performance on objects of varying size [19]. These design choices contributed to improved speed–accuracy behavior on common benchmarks, while also highlighting limitations such as sensitivity to precise localization at stricter IoU thresholds.

The broader literature positions YOLO among canonical one-stage detectors and contrasts it with region-based families (Fast/Faster R-CNN), particularly in application domains where dense scenes, small targets, and scale variation are known. For instance, object detection in high-resolution remote sensing imagery frequently emphasizes scale-adaptive features and robust localization under complex backgrounds—conditions that stress standard detection pipelines and motivate careful architectural choices [20].

Recent implementations extend YOLO from a detector into a unified framework that supports multiple computer vision tasks [22]. Ultralytics documentation emphasizes that the same ecosystem provides training workflows for detection, instance segmentation, oriented bounding boxes, pose estimation, and tracking, reflecting a shift from “detector-only” pipelines to configurable multi-task tool chains. In this context, YOLOv8 is presented as a free design model that can improve detection efficiency and accuracy, and it is positioned as a broadly applicable model for real-world deployment [21]. This means that it is possible to customize the trained model to identify different objects from the COCO database.

YOLO backbones combine feature extraction with multiple modules and libraries to achieve real-time performance [27]. Complementary Ultralytics [21] materials further describe architectural trends such as decoupling classification and regression components and introduce updated building blocks. This modernization is relevant for segmentation measurement (for instance, the hull-height extraction done in this paper) because instance segmentation provides pixel-level object delineation rather than only rectangular localization. Ultralytics’ segmentation task documentation explicitly frames segmentation as detecting and outlining objects in images, which aligns conceptually with methodologies that estimate physical dimensions from object contours after camera calibration or reference scaling.

The selection of YOLOv8 as the segmentation backbone for this work was based on several practical and technical considerations. At the time of system development, YOLOv8 represented a well-established and widely deployed framework, with extensive community support, comprehensive documentation, and readily available troubleshooting resources, factors that are particularly relevant for operational deployments in real port environments where system reliability and maintainability are critical requirements.

From a technical standpoint, comparative tests were conducted across multiple models within the YOLO family, and YOLOv8 consistently achieved a slightly superior segmentation performance for the specific task of hull contour delineation. While more recent architectures such as YOLOv9, YOLOv11, and transformer-based detectors like RT-DETR have since been introduced, the performance improvements they offer for this specific application do not currently justify the architectural modifications and retraining effort that a migration would require. Similarly, foundation models such as SAM (Segment Anything Model) [28] could, in principle, be adapted for hull segmentation. However, their integration would necessitate substantial changes to the geometric measurement pipeline and inference architecture, which are beyond the scope of the current work.

It is acknowledged that as newer models mature and their integration costs decrease, future iterations of this framework may benefit from exploring alternative segmentation architectures. For the present system, YOLOv8 provides a well-validated, efficient, and practically deployable foundation that meets the accuracy and stability requirements demonstrated in the results.

### 2.3. Camera-Based Draft-Reading Methods

The last decade has seen a rapid shift from purely manual draft observation toward camera-based and automated systems, driven by the need for safer, faster, and more objective draft surveys. Comprehensive reviews of ship draft observation technologies identify image recognition as the most promising alternative to human visual inspection, particularly when combined with unmanned aerial or surface platforms to access all six draft marks without exposing surveyors to hazardous conditions. Compared with contact sensors, camera-based approaches are non-intrusive, can be deployed temporarily on the quay or on small craft, and inherently provide a visual record that can be audited later, which is attractive for commercial and legal dispute resolution [29].

Early work focused on handcrafted image-processing pipelines that emulate the manual reading procedure by (i) locating the draft marks and their digits, (ii) locating the instantaneous waterline, and (iii) computing the draft by geometrically relating the waterline to the scale. A frequently cited line of research employs morphological operators, thresholding, and edge detection to isolate the high-contrast draft digits and the water–hull boundary. In an influential optical-engineering study, Tsujii et al. [8] propose a five-stage method comprising draft mark segmentation, digit recognition, waterline extraction, waterline estimation over time, and final draft calculation. Draft marks are first enhanced using white and black top-hat transforms with circular structuring elements sized just above the character stroke width, followed by local thresholding and removal of small noise regions, which dramatically improves segmentation compared with global Otsu binarization.

Zhang et al. [30] propose a computer vision-based system for automated ship draft measurement that integrates a climbing robot for image acquisition with image processing techniques, including morphological preprocessing, neural network-based character recognition, and color-based waterline segmentation. The method achieves a reported measurement accuracy of approximately 1 mm, representing a significant improvement over manual readings, while also reducing susceptibility to environmental factors such as wave motion and variable lighting and enabling repeatable, auditable measurements through automated data storage. However, the system still requires a human operator to board the vessel to deploy and operate the climbing robot, which introduces practical limitations in terms of safety, operational disruption, and access restrictions in regulated port and customs areas. In contrast, the framework proposed in this paper requires no human intervention during measurement, operating entirely from fixed quay-side cameras without any personnel boarding the vessel.

Waterline extraction in Tsujii et al. [8] is performed via Canny edge detection applied to each frame and subsequent selection of edge pixels near the expected waterline region; temporal accumulation and median filtering across frames are then used to suppress outliers due to wave motion and rain streaks. The authors explicitly tune their post-processing to mimic professional surveyors. Draft readings are computed for every frame, median filtering removes spurious extremes, and the final draft is taken as the mean of local maxima and minima within two standard deviations of the time-series mean, reflecting how surveyors visually average over wave crests and troughs. Experiments in a towing tank and in real harbor scenes report draft errors below 1 cm, even in heavy rain (about 10 mm/h), demonstrating the viability of purely image-based measurement under controlled conditions.

Complementary work emphasizes robustness to camera–target distance, again using morphological operations, thresholding, and robust regression to stabilize waterline estimation at different zoom levels [9]. In parallel, Ferreira et al. [10] develop a system focused on character recognition for automatic draft survey, in which camera images of the hull are processed to isolate the draft digits, which are then classified using pattern-recognition techniques to recover the numeric scale value. These systems show that relatively simple computer vision pipelines can achieve sub-centimeter accuracy in calm or moderately disturbed waters, but they remain sensitive to illumination changes, hull rust and fouling, complex reflections, and large waves [29].

To increase robustness under real-world conditions, more recent methods combine classical image processing with deep neural networks. Wang et al. [12] propose a ship draft line detection algorithm in which Harris corner detection, Canny edges, and the Hough transform are used to determine the straight water surface, while a convolutional neural network (CNN) performs digit recognition in the cropped draft-mark region. Tested on videos from various port scenes, the average discrepancy between automatic and professional manual readings is below 0.01 m, with occasional larger differences (>0.01 m) happening only in large wave cases where even manual readings are uncertain. This hybrid architecture preserves the interpretability of geometric waterline detection while leveraging deep learning where it is most beneficial, namely digit classification under diverse font, rust, and occlusion conditions [12].

Wei’s [29] review further discusses a system based on an improved YOLOv3 detector that directly predicts the approximate waterline position and identifies draft features in pre-processed images. In static-water scenarios, this method achieves high accuracy, outperforming earlier segmentation-based approaches; however, the authors note that no test data were collected for wave heights above 0.50 m, leaving robustness under heavy swell unresolved.

The current state of the art is represented by end-to-end deep learning systems that jointly handle draft scale recognition, waterline segmentation, and draft depth regression. Li et al. [13] propose a two-branch architecture combining a lightweight U^2^-NetP semantic-segmentation network, enhanced with coordinate attention and a YOLOv5n detection head for draft characters. The segmentation network partitions the image into background, hull, and water regions with a mean intersection-over-union of 96.47%, while YOLOv5n achieves a mean average precision (mAP) of 98% for character detection. By translating pixel distances into physical heights using the known 0.10 m character size, the system attains an average draft-reading error of 0.005 m and achieves “millimeter-level” precision across challenging conditions including large waves, floating obstacles, water traces along the hull, tilted characters, and heavily rusted scales.

Qu et al. [14] extend this paradigm by introducing an explicit multi-task learning framework for vessel draft reading. Their network simultaneously performs draft-scale recognition via object detection and vessel/water segmentation to localize the waterline. A specially designed loss function emphasizes pixels near the waterline to refine segmentation quality, and prior knowledge about the spatial distribution of draft scales is used for post hoc correction when characters are partially occluded or corrupted by stains. Draft depth is then estimated adaptively: when multiple scales are visible, the system uses the relative vertical distances between recognized scales and the waterline; when only a single scale is reliable, it switches to a character-height-based formula that leverages the known physical height of the draft numerals. Extensive experiments on a dedicated dataset covering draft mark detection, scale recognition, vessel/water segmentation, and depth estimation show that this framework improves both accuracy and robustness to hull stains relative to conventional OCR-based methods, while remaining computationally efficient enough for deployment on modest GPU hardware.

Zhang et al. [15] push performance further by exploiting multispectral imaging. Recognizing that RGB images are highly susceptible to reflections and color variations on the water surface, they acquire and annotate a dataset of 524 paired RGB and near-infrared (NIR) images of ship drafts and propose a dual-flow backbone (BIF) that fuses spectral information from both modalities. When integrated into an otherwise standard YOLOv8 detection head, the BIF backbone yields a draft-detection mAP of 99.2%. When paired with the UPerNet segmentation head for waterline detection, the mean IoU reaches 99.3%. Overall draft-reading error remains below ±0.01 m, illustrating how multispectral information can mitigate environmental interference such as specular reflections and variable water color.

Wei’s work [29] positions these deep learning approaches as the most likely successors to human visual inspection, especially when combined with unmanned vehicles (UAVs or unmanned boats) that can safely acquire images under diverse conditions while reducing labor costs. However, the review also highlights persistent challenges. For example, existing systems are typically validated under limited wave heights and lighting conditions, their performance in ice, strong swell, or heavy obstruction remains under-documented, and practical deployment must address the risks of tampering with image data or model outputs, which have implications for insurance and regulatory oversight.

Although not directly measuring draft scales, related work on imaging-based deadweight tonnage (DWT) estimation underscores the broader potential of vision systems for non-contact cargo assessment. Hou et al. [31] employ a rotation-aware vessel detector (RITSD) on high-resolution satellite images to obtain rotated bounding boxes for ships of various types and then regress DWT as a nonlinear function of the product of vessel length and breadth. The method achieves mean absolute percentage errors mostly below 7.7% across multiple vessel categories, suggesting that global cargo throughput and load management can, in principle, be monitored from remote sensing. While this approach operates at an entirely different spatial scale than camera-based draft reading, it illustrates the same trend. Computer vision is increasingly being used to infer mass parameters from images, complementing or partially replacing traditional hydrostatic calculations.

**Figure 2:**
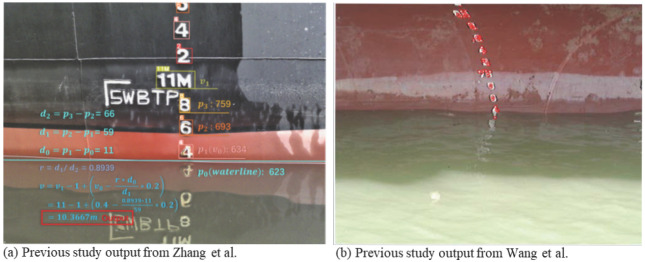
Examples of camera-based draft reading methods from the literature. (**a**) Adapted from Zhang et al. [15]. The vertical distance between each character, then determine the integer digit of the readings, calculate the decimal place of the readings via the vertical position of the waterline, and finally calculate the final readings with a correction factor. (**b**) Adapted from Wang et al. [12]. A Ship Draft detection using Hough transform on the contour map.

In the literature, as mentioned, SDS is done by using the draft marks. Figure 2 shows two examples found in the literature to clarify the difference between the literature and the proposed system.

Overall, the literature on camera-based and automated draft reading methods reveals a clear trajectory from early morphological and edge-based algorithms capable of centimeter accuracy in controlled environments, through hybrid systems combining geometric processing with CNN-based OCR, to contemporary deep, multi-task, and multispectral architectures under a wide range of conditions [12,15]. Despite these advances, full replacement of manual draft surveys will require further evidence of robustness in different sea states and complex operational contexts, standardized datasets and evaluation protocols, and governance frameworks that ensure data integrity and regulatory acceptance.

## 3. Methodology

The methodology of the proposed automated draft-survey system is organized into two complementary components. Section 3.1 describes the data transmission architecture, which enables reliable and secure transfer of images from edge devices operating at the berth to a local server and subsequently to a remote processing server. Section 3.2 presents the computational framework, detailing the geometric method for estimating hull height from static camera images. Together, these two components form an integrated pipeline that ensures continuous data flow, computational reliability, and operational scalability for maritime environments.

### 3.1. Data Transmission

The system is designed to operate in port environments where network reliability, interference, and physical obstructions can compromise conventional wireless communication paths. To guarantee the security of the data and consistent image transfer from the acquisition devices to the processing infrastructure, a dedicated 4G network is deployed using proprietary antennas, routers, and communication modules installed within the port facility.

**Figure 3:**
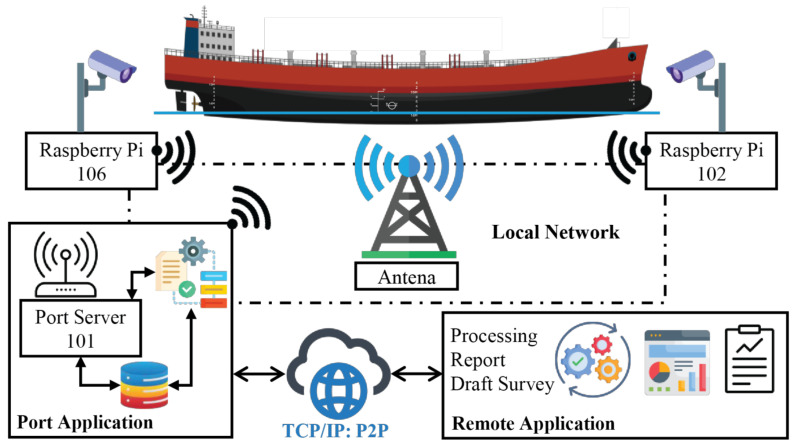
Model conceptualization.

Figure 3 represents the concept of data flow within the quay side in the terminal. Each imaging device, a fixed-position camera mounted on the quay structure, is equipped with an embedded device that contains a Raspberry Pi and a communication unit that transmits acquired images through the local private 4G network. Unlike public cellular networks, which may experience congestion or variable signal availability, the private configuration ensures dedicated bandwidth allocation, low latency, and stable coverage across the operational region of the berth.

Once images are captured, they are transmitted to a local edge server positioned within the same network infrastructure. This local server functions as the first aggregation layer and provides several advantages:

- Low-latency buffering, preventing data loss during transient network fluctuations.
- Redundancy management, ensuring that corrupted or incomplete frames are re-requested or discarded.
- Pre-processing, allowing the system to prioritize cameras or viewpoints as needed.
- Secure isolation, ensuring that raw data never leaves the port perimeter without passing through internal validation layers.

The system is organized into two main layers: a local private 4G network layer and a remote access layer, connected through a dedicated encrypted internet connection.

At the local level, two imaging devices, designated as source 102 (bow-side camera) and source 106 (stern-side camera), are independently and simultaneously deployed at fixed positions along the quay. Each device consists of a Raspberry Pi 4B single-board computer coupled with a high-resolution camera module and a 4G communication modem, as described in detail in Section 4. Both devices transmit captured images continuously and independently through the local private 4G network to a central antenna unit (eNodeB), which manages bandwidth allocation, signal routing, and network coverage across the operational area of the berth.

The central antenna forwards all incoming image streams to the Port Server (101), which functions as the primary processing node of the system. The Port Server hosts the Port Application, a software platform developed specifically for this framework, responsible for: (i) collecting and consolidating image streams from all connected camera devices; (ii) automatically identifying bow and stern viewpoints through the deployed segmentation models; (iii) executing the YOLOv8 instance-segmentation models and the geometric hull-height calculation pipeline described in Section 3; and (iv) storing timestamped results for subsequent analysis, reporting, and draft-survey support. All primary segmentation and measurement processing is performed locally on the Port Server, ensuring low-latency operation and data sovereignty within the port perimeter.

The Port Server is connected to a Remote Application via a dedicated encrypted TCP/IP peer-to-peer (P2P) internet connection. This connection serves two purposes. First, it enables authorized personnel to remotely access the system for monitoring, data collection, configuration, and maintenance, without requiring physical presence at the port. Second, the Remote Application mirrors the full processing capabilities of the Port Application and serves as a failover node: in the event that the Port Server becomes temporarily unavailable, the Remote Application can independently reprocess image data and resume hull-height estimation, ensuring the continuity of monitoring operations. All applications and software components described above were developed by the authors as part of this framework.

**Figure 4:**
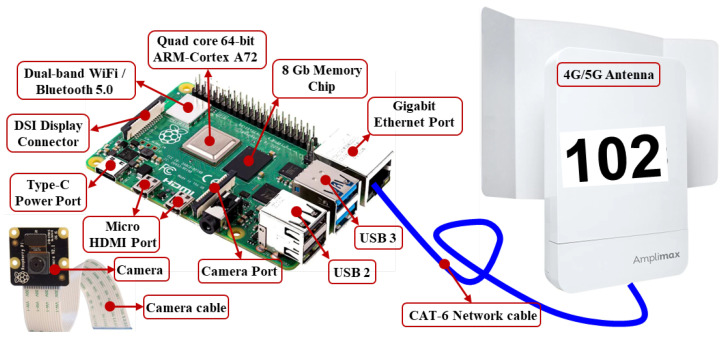
Raspberry Pi 4B mount representation.

Figure 4 shows the edge device used to capture images from both the bow and stern side of the ship. Primarily, this device is used to capture images with high resolution, ensuring that pre-processing is performed and the image is suitable for transmission. Aligned with the image description, Table 1 shows the specifications of the edge devices of each component.

After local processing, the images are securely forwarded from the edge server to a remote server via an encrypted Internet connection (secure tunneling protocols, peer-to-peer (P2P)). This second server hosts the segmentation and measurement algorithms, which use GPU acceleration and high-performance computing resources. Separating acquisition, buffering, and processing in this way enables the system to scale to a larger number of cameras while reducing the computational burden on edge devices.

To maintain synchronization and system integrity, the metadata accompanying each image includes a timestamp, camera/source ID, and network signal quality indicators, facilitating later quality control and traceability. The architecture also supports asynchronous transmission: if connectivity is temporarily degraded, data from each device are stored and processed locally until the connection is restored and link quality improves. This design ensures that no data is permanently lost and that processing remains robust even under challenging maritime network conditions.

This architecture establishes the data backbone required for the automated draft-survey framework proposed in this paper, ensuring that high-resolution imagery flows reliably from acquisition points to processing servers with the least possible data loss, and forms the foundation upon which the computational techniques operate.

### 3.2. Calculations Based on Segmentation and Geometric Measurements

This paper proposes a computer vision draft-survey method that does not rely on detecting draft marks. Instead, the system estimates the emergent hull height from segmented hull contours and converts pixel distances to metric units using a nearby bollard of known height as a reference object. The workflow consists of four main stages: (i) camera setup and image acquisition; (ii) image-based calibration using the bollard; (iii) hull segmentation using an instance-segmentation model (YOLOv8); and (iv) geometric extraction of hull height and pixel–meter conversion.

Instance segmentation with polygon-level masks is required for this approach because bounding box representations would include background pixels and surrounding port infrastructure, preventing reliable identification of the true hull boundary necessary for the geometric height extraction pipeline described in this section.

The system assumes that each vessel side is monitored by a static camera (for example, mounted on a quay or terminal structure) with a fixed intrinsic and extrinsic geometry during a survey campaign. For each camera, images are collected at several epochs and from multiple viewpoints around the bow and stern, but the camera itself remains static during processing for a given configuration.

For each camera configuration, a sequence of images is captured while the vessel is moored and stationary (or semi-stationary) alongside the berth. Images are stored in separate folders corresponding to each camera-source ID (“102” for bow and “106” for stern), and a dedicated and customized YOLOv8 segmentation model is associated with each configuration, reflecting differences in viewpoint, lighting, and hull appearance.

A defining component of this methodology is its bollard-based metric calibration, which replaces conventional scale derivation methods such as reference distances or camera calibration grids. Because the bollard is located physically close to the hull region being measured, and because both objects lie approximately in the same depth plane, the bollard provides a highly localized scale factor linking the image to the real world.

To formalize the measurement pipeline, let $P_{k}=\left\{\left(x_{j}^{\left(k\right)},\;y_{j}^{\left(k\right)}\right)\right\}$ denote the *k*-th segmented hull polygon, where $j=1,\ldots ,n_{k}$ indexes the polygon vertices and *k* indexes the detected hull instances in a given image. Let $\left(x_{1},y_{1}\right)$ and $\left(x_{2},y_{2}\right)$ denote the endpoints of a polygon edge, $x_{line}$ the horizontal coordinate of the vertical measurement line, t∈[0,1] the linear interpolation parameter along a polygon edge, $y_{int}$ the vertical coordinate of the intersection between the measurement line and a polygon edge, and $y_{bottom}$ the vertical pixel coordinate of the lowest detected hull point. In image coordinates, the vertical axis increases downward, so larger *y* values correspond to lower positions in the image.

Let the real bollard height be $H_{b}$ (meters), and let the manually annotated bollard polygon have pixel coordinates $\left\{\left(x_{i}^{\left(b\right)},\;y_{i}^{\left(b\right)}\right)\right\}$, where *i* indexes the bollard polygon vertices. The pixel height of the bollard is defined in Equation (1).

(1) $$h_{b}^{px}=\max_{i}\;y_{i}^{\left(b\right)}\;-\;\min_{i}\;y_{i}^{\left(b\right)}$$

Equation (2) defines the pixel-to-meter conversion scale:

(2) $$s=\frac{H_{b}}{h_{b}^{px}}\;\left[m/\mathrm{px}\right]$$

This calibration is performed exactly once per camera configuration, avoiding instabilities such as parallax distortions, asymmetric viewpoints, and dynamic trim and heel effects. Since the bollard is rigid, steady, and dimensionally known, it bypasses the need for waterline detection, which is often degraded by wave motion, reflection, and hull discoloration.

Following calibration, each image is processed using a YOLOv8 instance-segmentation model trained to detect the visible hull surface as one or more polygonal masks. Unlike the methods reviewed in Section 2 that isolate draft numbers or waterline edges, the present model focuses solely on the hull boundary, which remains visually stable even under variable environmental conditions. For each segmented hull polygon $P_{k}$, the system identifies the lowest visible hull point, corresponding to the maximum *y* pixel coordinate among all vertices (Equation (3)):

(3) $$y_{max}=\max_{j}\;y_{j}^{\left(k\right)}$$

Depending on the camera orientation, the horizontally appropriate extreme is selected (leftmost for left-view cameras, rightmost for right-view cameras), forming the point $\left(x_{line},\;y_{bottom}\right)=\left(x_{line},\;y_{max}\right)$, which defines a unique vertical measurement line perpendicular to the waterline plane.

The upper hull boundary along this line is found by computing all intersections between the vertical line $x=x_{line}$ and the polygon edges of the segmentation mask. For each polygon edge connecting $\left(x_{1},y_{1}\right)$ and $\left(x_{2},y_{2}\right)$, an intersection occurs whenever the segment spans the measurement line, as shown in Equation (4):

(4) $$\left(x_{1}-x_{line}\right)\left(x_{2}-x_{line}\right)\leq 0,\;x_{1}\neq x_{2}$$

The intersection coordinate is then obtained by linear interpolation along the edge (Equations (5) and (6)):

(5) $$t=\frac{x_{line}-x_{1}}{x_{2}-x_{1}},\;0\leq t\leq 1$$

(6) $$y_{int}=y_{1}+t\;\left(y_{2}-y_{1}\right)$$

where *t* is the normalized interpolation parameter representing the relative position of the intersection point along the edge. All intersections satisfying $y_{int}<y_{bottom}$ are considered candidates for the upper hull limit; the smallest such value defines the top point, expressed in Equation (7):

(7) $$y_{min}=\min\;\left\{y_{int}\;:\;y_{int}<y_{bottom}\right\}$$

The pixel height of the emergent hull (the freeboard segment observed by the camera) is then given by Equation (8):

(8) $$h^{px}=y_{bottom}-y_{min}$$

and the corresponding metric hull height is finally obtained by Equation (9):

(9) $$H=s\cdot h^{px}$$

This height represents a direct geometric estimate of the visible vertical hull segment at the measurement line, independent of draft marks or waterline location. In contrast with previous methods reviewed in Section 2, which rely on accurate waterline segmentation, digit recognition, or multi-task learning, the present approach entirely avoids these components.

An implicit assumption of the bollard-based calibration is that the bollard and the vessel hull lie at approximately the same depth plane relative to the camera. In practice, however, the camera-to-bollard distance and the camera-to-hull distance will differ, introducing a perspective-induced scale error that must be acknowledged and quantified.

Under a standard perspective projection model, the apparent size of an object in the image is inversely proportional to its distance from the camera. Let $d_{b}$ denote the horizontal distance from the camera to the bollard (meters) and $d_{h}$ denote the horizontal distance from the camera to the hull (meters). The pixel-to-meter scale factor *s* derived from the bollard at distance $d_{b}$ will differ from the true scale factor at the hull distance $d_{h}$ by a relative perspective error ϵ, defined in Equation (10).

(10) $$\epsilon =\left|\frac{d_{h}-d_{b}}{d_{b}}\right|$$

where ϵ is dimensionless and represents the fractional scale discrepancy introduced by the depth plane offset between the bollard and the hull. For the experimental configuration deployed at the Port of Santos, the camera-to-bollard distance is $d_{b}=82.3$ m and the camera-to-hull distance is $d_{h}=84.0$ m, yielding a perspective error described in (10).

(11) $$\epsilon =\left|\frac{84.0-82.3}{82.3}\right|\approx 0.021$$

This corresponds to a relative scale error of approximately 2.1%, which, for the maximum observed hull height of H=8.27 m, translates to an absolute metric uncertainty of approximately 0.17 m. This error magnitude is consistent with the operational objectives of the proposed system, which targets continuous monitoring of vessel loading trends rather than centimeter-level draft-survey accuracy. Furthermore, since both the bollard and the hull are fixed structures observed from a static camera throughout the entire monitoring campaign, this perspective error remains constant across all measurements and introduces a systematic rather than random bias. As a systematic offset, it affects the absolute scale of the hull-height estimates but does not influence the relative trends, slopes, or temporal consistency of the measurements, which are the primary indicators used for loading state assessment in this work.

The bollard used as the metric reference in this work is a fixed and permanent port infrastructure element at the Port of Santos. As part of standard port surveying practice, all bollards are georeferenced structures whose positions and physical dimensions are formally recorded and maintained by the port authority. The bollard height $H_{b}$ used in the calibration is, therefore, not an ad hoc field measurement but a certified dimensional value obtained from official port infrastructure records, ensuring that the scale factor *s* derived from Equation (2) is based on a reliable and traceable reference. This eliminates the measurement uncertainty that would otherwise be associated with manual height estimation of the reference object, and differentiates the proposed calibration approach from methods that rely on improvised or uncertified reference targets.

The algorithm implemented, described in Algorithm 1, executes automatically across all images, generating both annotated images and a CSV (Comma Separated Values) file containing the pixel and metric hull heights for every hull instance. The process includes YOLOv8 forward inference; mask filtering; geometric extraction of the lowest hull points; vertical line intersection and visualization; height computation; and metric conversion.
**Algorithm 1** Hull Height Estimation Procedure  1:**for** each camera source **do**  2:   Load the segmentation model  3:   **for** each image **do**  4:     Run segmentation to obtain hull masks  5:     **for** each hull polygon **do**  6:        Identify the bottom-most hull point  7:        Select $x_{\mathrm{line}}$ based on view orientation  8:        Compute intersections with polygon edges  9:        Determine $y_{\min}$ and $y_{\mathrm{bottom}}$10:        $h_{px}\leftarrow y_{\mathrm{bottom}}-y_{\min}$11:        $H\leftarrow s\cdot h_{px}$12:        Record results and annotate the image13:     **end for**14:   **end for**15:**end for**

It is important to note that the framework presented in this section was designed and validated with bulk carrier operations as the primary scope. The segmentation models are trained on imagery specific to this vessel class, and the camera placement strategy assumes berth configurations typical of bulk terminals. Nevertheless, the modular architecture of the system, in which segmentation models, camera configurations, and network components are independently deployable, is intended to facilitate future adaptation to other vessel types and port environments, as discussed in Section 6.

## 4. Experimental Setup and Field Tests

### 4.1. Experimental Site: Port of Santos, Brazil

**Figure 5:**
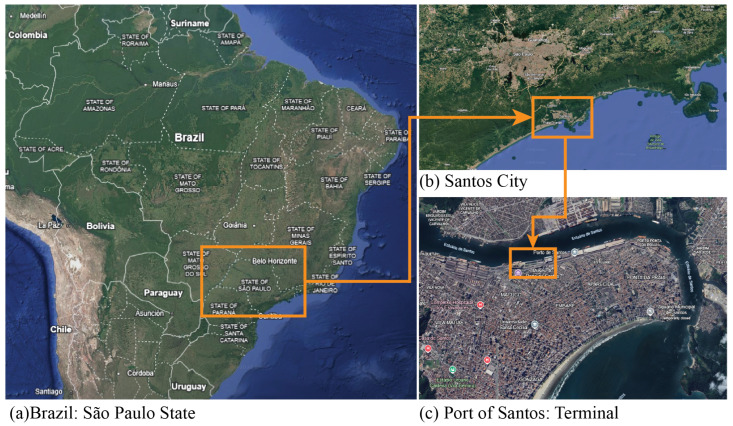
Experiment location.

Field experiments were conducted at the Port of Santos, Brazil, the largest port complex in Latin America and a critical hub for bulk cargo exports. Due to its high operational throughput and strict safety and access regulations, the port represents a challenging but realistic environment for validating automated draft-survey technologies. The experiments were carried out in cooperation with the Port Authority of Santos (SPA). Figure 5a shows the global location of Port of Santos, in São Paulo state, more specifically Santos City. Figure 5b indicates the location of Port of Santos and the position of the terminal (Figure 5b) where the experiment was carried out.

The selected test area was the berth adjacent to Warehouse 39, where bulk carriers operated by ADM do Brasil routinely load grain cargo. This berth was chosen for several reasons: (i) frequent bulk loading operations, (ii) clear visibility of the hull from the quay, (iii) reduced vehicular traffic due to its position at the end of the pier, and (iv) proximity to densely populated urban areas, where operational transparency and environmental monitoring are of particular social relevance. However, since this wharf is located adjacent to cruise ship terminals, loading operations may occasionally be disrupted by cruise ships.

**Figure 6:**
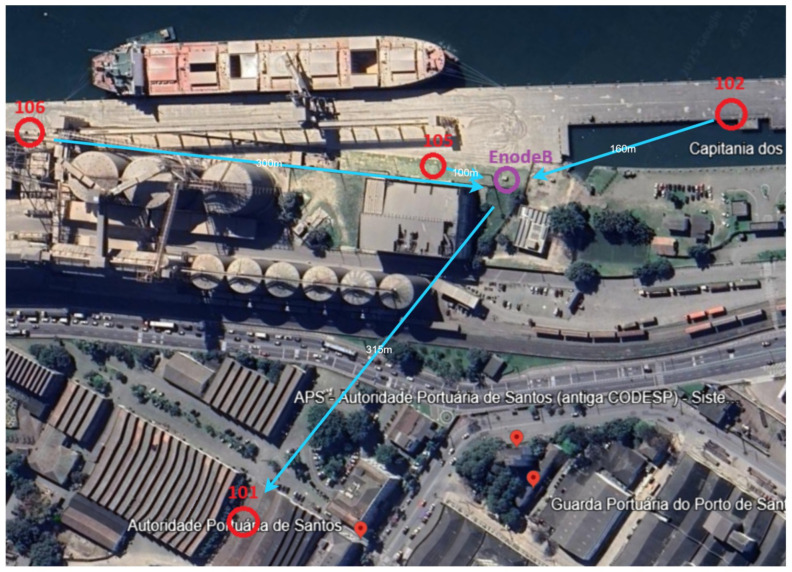
Equipment location at Port of Santos.

Figure 6 shows the exact locations of each device necessary for this research. Each component (cameras, antennas, and modems) was strategically positioned to take the best angle of multiple vessel sizes and configurations. Note that all camera devices, shown in the Section 3, are connected to the “EnodeB”, which makes the appropriate connection to the local server, located at the SPA office. SPA officers can check and configure the system at their convenience and when necessary.

The physical layout of the experimental area includes a wide quay, fixed loading infrastructure (silos, conveyors, and ship loaders), and sufficient longitudinal clearance to observe the vessel hull from bow to stern. Based on technical inspections and site visits performed in December 2024, two camera kits were deployed at fixed locations along the quay, providing complementary viewpoints of the vessel. The camera and antenna components were designated as 102 and 106 devices, each serving a distinct observational role:

- Device 102 was positioned to capture the bow region of the vessel;
- Device 106 monitored the stern region, enabling comparative analysis between fore and aft hull heights.

By comparing the hull-height measurements obtained from devices 102 and 106, it is possible to infer longitudinal trim variations during loading operations, consistent with the hydrostatic principles discussed in Section 2.

Camera setup was constrained by port safety regulations, existing electrical infrastructure, and the need to avoid interference with terminal operations. As a result, installations were performed with the assistance of port maintenance teams using elevated platforms, and camera positions were selected to balance measurement accuracy with operational feasibility.

All camera devices were integrated into the private 4G communication architecture described in Section 3. Each camera and modem cluster functioned as a client device, transmitting captured images to a mobile local server placed in a dedicated position (EnodeB) within the terminal area. This trailer accommodated networking equipment, power units, and communication antennas, allowing rapid deployment and simplified maintenance without permanent port infrastructure modifications.

The local server aggregated images from all camera clusters using a TCP/IP-based protocol designed to ensure reliable and error-free transmission. From this local node, images were forwarded via a secured Internet connection to a remote processing server, where the segmentation and geometric measurement algorithms described in Section 3 were then executed. This two-stage transmission strategy was adopted to address the known limitations of port environments, including radio-frequency obstruction by the terminal permanent infrastructure, metallic structures, and ship superstructures.

**Figure 7:**
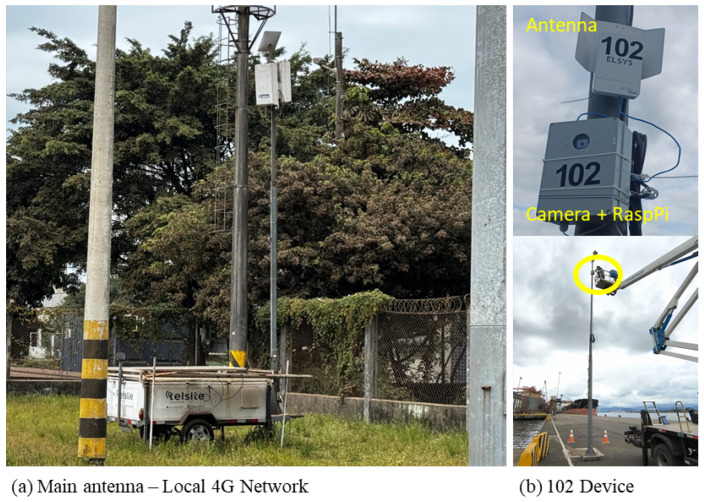
Antenna and 102 device installation.

Figure 7a shows the position of the main antenna near the quay (shown as EnodeB in Figure 6). Note that this device was specifically built to be mobile to accommodate 4G infrastructure, solar panels, and batteries. Figure 7b shows the installation of the 102 camera device with its antenna.

### 4.2. Data Collection

Image acquisition was conducted over multiple days and operational cycles, capturing vessels in static mooring conditions during different stages of loading. Cameras operated at a fixed resolution and captured time-stamped images at regular intervals, generating a dataset that reflects realistic variations in lighting, weather conditions, hull appearance, and background clutter. During the experimental campaign, images were collected under diverse environmental conditions, including:

- Clear and cloudy skies;
- Dry and wet quay surfaces following rainfall;
- Variable water reflectance;
- Different vessels passing nearby or support boats;
- Partial occlusion of the hull by port equipment.

These conditions were intentionally retained in the dataset to evaluate the robustness of the segmentation-based hull measurement approach under operationally relevant disturbances, rather than restricting evaluation to ideal laboratory conditions. The primary objective of the experimental campaign was to validate the feasibility of segmentation hull-height estimation in a real port environment, without relying on draft mark visibility. Specifically, the experiments aimed to:

- Verify the stability of hull segmentation across different viewpoints and environmental conditions;
- Assess the consistency of hull-height measurements derived from repeated images captured by the same camera;
- Evaluate the comparative behavior of bow and stern measurements for detecting trim-related variations;
- Demonstrate reliable end-to-end operation of the data transmission and processing pipeline.

Rather than benchmarking against a draft mark value, which is itself subject to uncertainty in manual surveys, the experimental evaluation focused on repeatability, continuity, and operational robustness, aligning with the long-term objective of continuous draft monitoring rather than discrete survey replacement.

### 4.3. Labeling and Training Parameters

**Figure 8:**
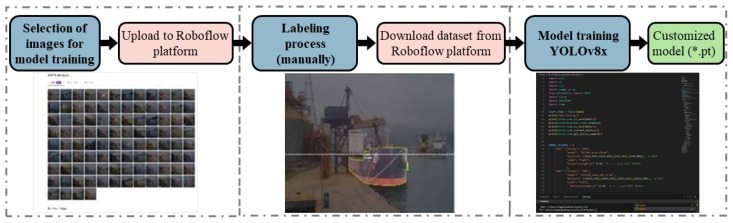
Labeling process.

The performance of instance-segmentation models such as YOLOv8 is highly dependent on the quality and representativeness of the labeled training data. In this study, a dedicated labeling process was designed to accurately represent the visual characteristics of ship hulls observed in real port environments, as described in Section 4. The objective of the labeling stage was not to identify draft marks or waterlines, but rather to produce precise hull contours suitable for subsequent geometric height extraction. Figure 8 describes this process.

The initial dataset was constructed from images captured by the camera devices installed at the Port of Santos, devices 102 and 106, which observed the bow and stern regions, respectively. Images were selected across multiple days and operational states to ensure diversity in (a) hull geometry and paint condition; (b) illumination (morning, midday, and late afternoon); (c) weather (clear, cloudy, post-rain); (d) quay surface reflectance; and (e) background clutter (cranes, conveyors, passing vessels). Table 2 shows an overview of all images used for training the YOLOv8 segmentation models. To train the bow-view model, 70% of all images were used for training, while 20% were used for validation and then 10% testing. On the stern side, the proportions used were 80%, 13% and 7%, respectively.

This diversity was intentionally preserved to improve the generalization capability of the trained model and to avoid overfitting to idealized conditions. Images in which the hull was fully occluded or severely distorted by motion blur were excluded, as they do not represent valid measurement scenarios.

Labeling was performed using the Roboflow platform [32], which supports polygon-based annotation compatible with YOLOv8 instance segmentation. A single semantic class, ship hull, was defined. This design choice reflects the methodological objective of extracting hull geometry only, rather than performing multi-class detection or draft-mark recognition.

Each image was annotated by manually drawing a closed polygon tightly following the visible outer contour of the hull, from the upper deck line down to the lowest visible hull point above the water. Particular care was taken to:

- Exclude water reflections and wake patterns;
- Avoid including quay structures, fenders, or mooring lines;
- Maintain continuity along curved hull regions near the bow and stern.

**Figure 9:**
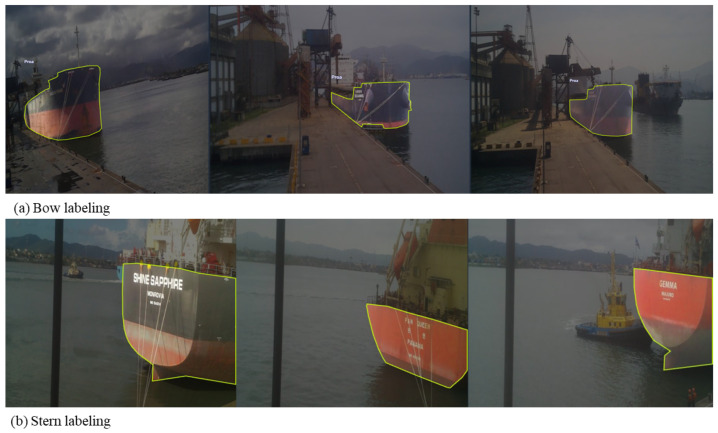
Example of labeling for training YOLOv8 segmentation model.

This polygon-based approach allows YOLOv8 to learn the full spatial extent of the hull rather than a bounding box approximation, which is essential for the geometric intersection method described. Figure 9 shows examples used to train the YOLOv8 model, shown in Figure 9a for bow-view images and in Figure 9b for stern-side view.

Following the labeling procedure described in the previous subsection, the annotated dataset was used to train YOLOv8 instance-segmentation models for hull detection. The same training configuration and hyperparameters were applied to models corresponding to both the bow and stern viewpoints, ensuring methodological consistency and enabling direct comparison of segmentation performance across different vessel sections.

The dataset was structured using the standard YOLOv8 configuration file (data.yaml), which explicitly defines the directory structure for training, validation, and test images to support supervised learning, hyperparameter monitoring, and unbiased performance evaluation. The use of a single semantic class (number of classes $\overset{-}{1}$) reflects the methodological focus of this study: segmentation of the ship hull as a geometric object rather than multi-class detection or draft mark recognition.

To improve model generalization under real port operating conditions, a data augmentation [33] strategy was applied during training. Table 3 shows the augmentation parameters that are important given the limited control over environmental factors such as lighting, weather, reflections, and background in port environments.

Model training was performed using the Ultralytics YOLOv8 framework. A relatively large number of training iterations (epochs = 1000) was selected to allow convergence under the combined effects of data augmentation and input resolution. Early stopping was enabled through a patience parameter of 100 epochs, ensuring that training terminated automatically if no improvement was observed on the validation set, thereby preventing unnecessary overfitting.

The input image size was set to 640 × 640 pixels, significantly higher than typical object-detection configurations, to preserve fine hull-boundary details critical for accurate geometric height extraction. Parallel data loading was enabled with 12 worker threads to maximize throughput during training.

The same training configuration was applied independently to the bow and stern datasets. This design choice ensured that any performance differences between models arose from specific visual characteristics rather than differences in hyperparameter tuning, consequently strengthening the validity of the comparative analyses presented in the Section 5.

Both bow and stern segmentation models were initialized from the YOLOv8x-seg weights pretrained on the COCO dataset [34], following standard transfer learning practice. Although COCO-pretrained weights provide general low-level feature extraction capabilities, the visual characteristics of ship hulls, including large uniform painted surfaces, complex geometric boundaries, variable illumination, and maritime background clutter, differ substantially from typical COCO object categories. Fine-tuning on the custom port dataset was, therefore, essential to adapt the pretrained backbone to the specific appearance and geometry of hull contours observed from quay-side viewpoints.

The SGD optimizer was used for both models with an initial learning rate of $lr_{0}=0.01$, a final learning rate ratio of $lr_{f}=0.01$, momentum of 0.937, and weight decay of 0.0005. A linear warmup was applied over the first three epochs with a warmup momentum of 0.8 and warmup bias learning rate of 0.1. The loss function comprised three components with fixed weights: bounding box regression loss ($w_{box}=7.5$), classification loss ($w_{cls}=0.5$), and distribution focal loss ($w_{dfl}=1.5$). Training used a batch size of four images with 12 parallel data loading workers and automatic mixed precision (AMP) enabled to optimize memory usage. An IoU threshold of 0.7 was applied during validation. It should be noted that while the optimizer and core hyperparameters were consistent across both models, the data augmentation parameters were tuned independently for each viewpoint to account for the distinct visual characteristics of bow and stern images, as reflected in the augmentation settings reported in Table 3.

The YOLOv8x-seg model employed in this work consists of 232 layers with 71,751,811 trainable parameters and a computational complexity of 328.8 GFLOPs, making it the largest and most capable variant in the YOLOv8 family. The network processes input images of a fixed spatial resolution $X\in R^{3\times 640\times 640}$, where the three channels correspond to the RGB color space and the spatial dimensions are standardized through letterbox resizing to preserve the original aspect ratio while padding to the target resolution.

## 5. Results

This subsection presents the training outcomes and segmentation performance of the YOLOv8 models developed for hull detection at the bow and stern viewpoints. As described previously, both models were trained using identical labeling strategies, data augmentation schemes, and hyperparameters, enabling a consistent and unbiased comparison of their behavior across distinct geometric perspectives.

### 5.1. YOLO Training Convergence and Loss Behavior

**Figure 10:**
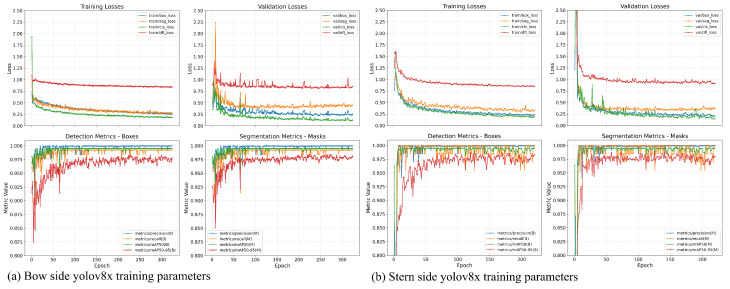
YOLO training metrics’ output.

Figure 10a shows the evolution of the training and validation losses for bounding box regression (box_loss), segmentation mask prediction (seg_loss), classification (cls_loss), and distribution focal loss (dfl_loss) for the bow model, while Figure 10b presents the corresponding curves for the stern model.

For both viewpoints, all loss components exhibit rapid initial decay, followed by a smooth convergence as training progresses. The segmentation loss decreases sharply within the first 50–100 epochs, indicating that the model quickly learns to delineate the hull contour. After this initial phase, loss values stabilize and gradually decrease, suggesting that later epochs are primarily refining boundary precision rather than correcting gross localization errors.

The close alignment between training and validation loss curves across all components indicates the absence of significant overfitting, despite the relatively long training duration (up to 1000 epochs). This behavior confirms the effectiveness of the adopted data augmentation strategy and early stopping mechanism, as discussed. Notably, the stern model exhibits slightly higher initial loss variability, which can be attributed to increased background complexity and partial occlusions commonly present in stern-view images.

Detection and segmentation performance can be evaluated using standard YOLOv8 metrics computed on the validation set, including precision, recall, and mean average precision (mAP) at different Intersection over Union (IoU) thresholds.

For both bow and stern models, precision and recall for bounding box detection (metrics/precision (B) and metrics/recall (B)) rapidly approach values close to 1.0, indicating highly reliable hull localization. Similarly, segmentation metrics (metrics/precision (M) and metrics/recall (M)) converge to near 1.0 values, confirming that the predicted masks closely match the annotated hull contours.

The mAP results further support this conclusion. Both models achieve values approaching 1.0 for bounding boxes and masks, demonstrating robustness under stricter IoU thresholds. This is particularly important for the subsequent geometric analysis, as small segmentation errors near the hull boundary could propagate into height measurement inaccuracies.

Minor fluctuations observed in early validation epochs are consistent with the application of mosaic and MixUp augmentations and disappear as training stabilizes. Overall, the quantitative metrics confirm that YOLOv8 provides accurate and stable hull segmentation for both viewpoints.

**Figure 11:**
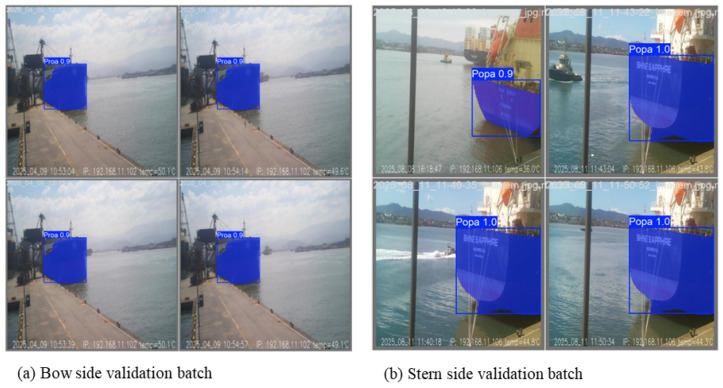
Validation batch with prediction.

Representative validation images for the bow and stern models are shown in Figure 11. In these examples, the segmented hull masks closely follow the visible hull geometry. The dataset used in training stages had multiple conditions, such as variable illumination and sky brightness, water reflections, weather, and moving vessels around, to increase accuracy and avoid overfitting.

The segmentation results remain stable across repeated images of the same vessel taken at different times, indicating that the model generalizes well beyond individual frames. This qualitative consistency complements the quantitative metrics and supports the suitability of the trained models for geometric processing.

The convergence behavior, segmentation accuracy, and consistent qualitative performance observed for both bow and stern models establish a reliable foundation for the hull-height calculation methodology. Because the proposed measurement approach relies on extracting precise hull contours and identifying extreme points along vertical lines, segmentation stability is more critical than raw detection accuracy itself.

### 5.2. Data Communication Performance and Transmission Behavior

**Figure 12:**
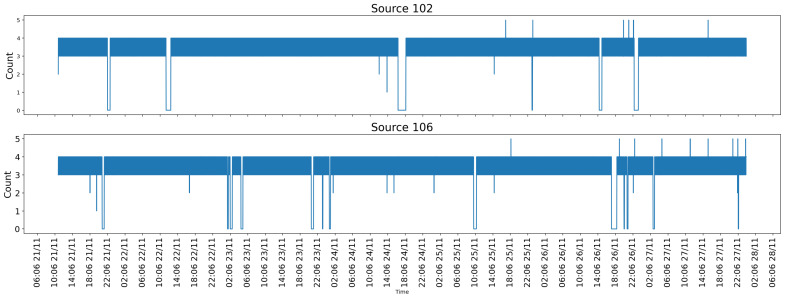
Temporal behavior of image transmission.

Figure 12 illustrates the temporal behavior of image transmission from the bow-side device (source 102) and the stern-side device (source 106) to the local server via the private 4G network described in the methodology. The time-series plots reveal generally steady transmission behavior under the network conditions. The occasional short-duration drops to zero observed reflect two distinct categories of disturbance. The first category corresponds to temporary electricity outages within the port infrastructure, which cause the edge devices to power off. In such cases, no buffering or retransmission is possible, and the affected frames are permanently lost. However, as these outages are attributable to external port infrastructure conditions rather than system design limitations, they are considered outside the scope of the proposed framework’s fault tolerance capabilities.

The second category corresponds to temporary network connectivity degradation, such as radio-frequency attenuation caused by metallic port structures or passing vessels. To handle these occurrences, each Raspberry Pi edge device is equipped with an internal buffering script that stores captured images locally during connectivity interruptions and automatically retransmits them to the Port Server once the network connection is reestablished. This mechanism ensures that no image data is permanently lost due to transient network disturbances.

Importantly, both categories of disturbance were infrequent throughout the 6.5-day experimental campaign and did not significantly affect the continuity or integrity of the hull-height calculation results.

To complement this time-series visualization, Table 4 summarizes the total volume of data collected and transmitted by each device over the experimental period. Both devices operated continuously for approximately six and a half days, starting and ending at nearly identical timestamps. Device 102 transmitted a total of 34,754 images, while device 106 transmitted 35,035 images, corresponding to average transmission rates of 3.68 images/min and 3.71 images/min, respectively. The close agreement between these rates demonstrates consistent acquisition scheduling and stable network performance across independent devices, confirming that the overall data availability was sufficient for reliable continuous monitoring.

For source 102, there were long intervals of steady transmission, typically three to four images per timestamp interval, indicating sustained connectivity. The source 106 device exhibits a similar overall transmission profile but with slightly higher variability, which is consistent with its stern-side placement. The stern environment is more frequently affected by wake turbulence, passing vessels, and metallic obstructions, all of which can momentarily degrade wireless signal quality. Nevertheless, the cumulative statistics confirm that these disturbances do not significantly impact long-term data availability.

Taken together, the time-series plots and the aggregate statistics demonstrate that the proposed communication architecture provides reliable, continuous, and sufficiently high-throughput image transmission to support long-duration monitoring and downstream processing. The consistent data rates and high image counts from both devices form a robust foundation for the hull-height calculations.

### 5.3. Hull-Height Calculation Results

Novaes Mathias et al. [35] suggested a timeline that is a conceptual and analytical construct that represents events and activities in a structured chronological sequence. It organizes discrete occurrences according to their temporal order and duration, thereby transforming individual records into a coherent process-oriented representation. By linking events through time, a timeline makes explicit not only what occurs, but also when it occurs and for how long, enabling a systematic understanding of sequences, dependencies, and transitions within a given system.

The importance of a timeline lies primarily in its capacity to support analytical clarity and comparability. Temporal structuring allows researchers and practitioners to identify patterns, bottlenecks, and inefficiencies that are not evident in isolated or static records. By visualizing or modeling durations between events, timelines enable the derivation of time-based indicators, such as waiting times, processing times, and total cycle durations, which are essential for performance assessment and benchmarking.

The scope of this research does not cover a complete timeline of vessel events. However, it is possible to automatically take some of the events suggested to build part of the timeline and understand the operation processes.

This experiment was done by monitoring the vessel KALLIOPI L, a dry bulk carrier (bulk cargo ship) categorized in the Panamax size class. This means that its maximum dimensions are suitable for transiting the original Panama Canal locks, which has historically been a key constraint for bulk shipping routes. It was built in 2001 and is presently active and sailing under the flag of Liberia with the IMO number 9233284, callsign A8GS8 and MMSI 636012640. The vessel’s main dimensions are approximately 225 m in length overall (LOA) and about 32 m in beam (width). The deadweight capacity (DWT) is around 76,500 tonnes, and the gross tonnage is roughly 39,994 (all public information found online). Table 5 shows the timestamps of events following the literature [35]; these events are taken through observation of the images and the ship’s statement of facts. It is important to highlight that not all items are filled because of the scope of this paper.

**Figure 13:**
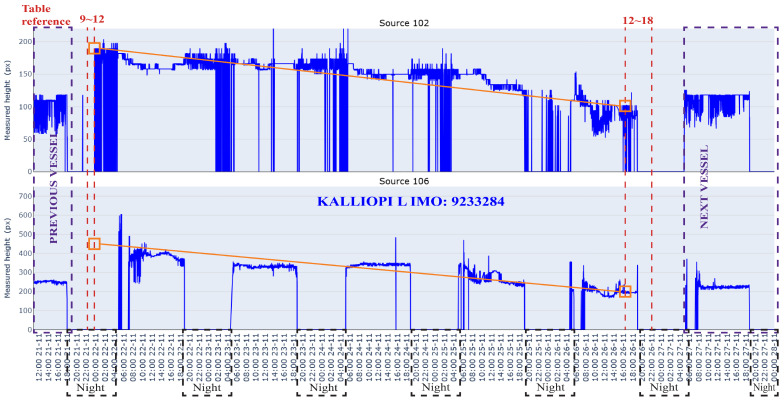
Model output of hull-height estimation.

Figure 13 shows the time series of hull-height estimates obtained from the bow-side camera (source 102) and the stern-side camera (source 106) during the experimental period. The hull-height measurements exhibit a clear decreasing trend over time, highlighted by the orange reference line. The maximum measured hull height is 190 pixels, corresponding to the first valid height detection, while the minimum value of 102 pixels is observed near the end of the cargo operation. This reduction in measured height is consistent with progressive cargo loading, which reduces the vessel’s freeboard. Periods with zero values correspond to intervals with no valid detection, primarily during nighttime, insufficient illumination, or network issues.

For source 106, a similar but lower-magnitude trend is observed. The maximum hull height measured for source 106 is 492 pixels, while the minimum value of 196 pixels is taken at the same time window as in source 102. Although the absolute magnitudes differ due to camera geometry, camera position, and local reference scaling, the similarity of the slopes across independent viewpoints suggests a coherent longitudinal loading process rather than measurement noise. This correspondence implies that the rate of change of the observed height, dH/dt, can be interpreted as a proxy for the vessel loading rate, with steeper slopes corresponding to a higher loading intensity.

The purple dashed rectangle on the left side shows the previous vessel operation (THOR CONFIDENCE), then our target vessel, KALLIOPI L, and on the right side, the next vessel in line to operate. The dashed red lines show the relative timestamps, presented in Table 5, where it is possible to compare the timestamps collected using the current system with the vessel’s statement of facts.

The results of the hull-height estimation measurements, as mentioned before, were obtained independently from the bow- and stern-side cameras, using the customized trained YOLOv8x segmentation model [22]. The results are analyzed in terms of temporal stability, consistency across viewpoints, and robustness under real port operating conditions. It is important to mention that while it is possible to have measurements with source 102 during nighttime, this behavior could not be seen with source 106 due to the camera’s position and illumination.

For both devices, hull height was computed image by image by extracting the vertical extent of the segmented hull contour and converting pixel distances to metric units using the bollard-based calibration. The resulting time series plots show a high degree of temporal continuity, with hull-height values remaining stable across consecutive images captured during static mooring conditions. The height from pixels to meters of the bow-side view (source 102) decreases from 8.27 m to 4.64 m, while the stern-side view (source 106) goes from 7.98 m to 3.98 m over approximately the same time window.

In the bow-side view, the predicted hull height exhibits minor fluctuations around a well-defined mean value. These variations are small relative to the absolute hull height and are primarily attributable to: (i) camera viewing-angle alignment relative to the vessel’s hull, giving a slight perspective sensitivity near curved bow geometry; (ii) residual segmentation noise at the hull boundary; and (iii) small wave-induced changes in the visible waterline interface. Despite these factors, no abrupt discontinuities or systematic drift are observed, indicating that the segmentation masks and the geometric extraction procedure remain stable over time.

Similarly, the stern side produces consistent hull-height estimates, although with slightly higher short-term variability. This behavior can be explained by more complex visual elements such as propeller visual turbulence and background vessel traffic. Nevertheless, the measured hull height remains confined within a narrow range, confirming the robustness of the method even under some visual disturbance.

Intervals where the measured hull height equals zero correspond to periods with no valid detection, which occur predominantly during nighttime when ambient illumination is insufficient. In these cases, the YOLOv8 segmentation model correctly produces no hull masks, and the system intentionally suppresses measurement output to avoid unreliable estimates.

Quantitatively, over the monitoring period of about 6.5 days, each device transmitted more than 34,000 images at an average rate of approximately 3.7 images per min. Valid hull-height measurements were obtained during the majority of daylight hours, while zero-value intervals were confined to nighttime or low-visibility conditions. This behavior confirms that the system provides continuous and repeatable measurements whenever visual conditions permit, while safely disabling measurement under inadequate illumination.

## 6. Discussion

A key objective of the experimental campaign was to assess whether hull-height measurements derived from independent viewpoints are mutually consistent. The results show that bow and stern hull-height estimates follow similar temporal trends, with differences that can be interpreted in the context of vessel trim rather than measurement instability.

In periods where the vessel loading condition remains unchanged, both bow and stern measurements remain approximately constant, indicating that the system is not sensitive to camera-specific noise or transient background changes. When small differences between bow and stern values are observed, these differences are persistent over time rather than random, suggesting a physical origin related to longitudinal trim rather than algorithmic error.

This behavior aligns with classical draft-survey principles, described in Section 2, where differences between forward and aft draft readings are used to infer trim. Although the present method does not directly measure draft, the consistency between bow and stern hull-height estimates supports its suitability for relative trim monitoring and continuous observation of vessel loading state.

An important outcome of the results is the repeatability of the hull-height measurements under repeated observations of the same vessel from the same camera. For both devices, consecutive frames yield highly similar height estimates, and repeated measurements taken minutes apart show no systematic bias.

The absence of frequent large outliers or discontinuities confirms that the vertical line intersection strategy used to extract hull height is robust to minor segmentation boundary variations. This is particularly relevant given that the measurement relies on identifying extreme points of the hull contour, which could otherwise be sensitive to segmentation artifacts.

Moreover, the use of a local physical reference (bollard) for pixel-to-meter conversion contributes to measurement stability by reducing scale errors associated with camera perspective and distance variations. As a result, the observed hull-height fluctuations are dominated by environmental and physical factors rather than calibration uncertainty.

Importantly, these results were obtained under real operational conditions at the Port of Santos, including variable lighting, background activity, and water surface dynamics. This reinforces the practical viability of the approach and differentiates it from laboratory-based or ideal environment computer vision demonstrations.

The hull-height calculation results confirm that segmentation measurement can deliver reliable and consistent outputs suitable for operational maritime environments. The agreement between bow and stern measurements, together with their temporal stability, validates the methodology presented in Section 3 and complements the segmentation performance presented in this paper.

Although the experimental validation presented in this work was conducted at the Port of Santos using a Panamax bulk carrier, the proposed framework is designed with operational flexibility in mind and is not inherently restricted to this specific site or vessel class. Several aspects of the system architecture contribute to its generalization.

From an infrastructure standpoint, the system requires only a standard electrical power supply for each edge device, with no dependency on permanent port installations or specialized quay structures. A solar panel and battery-based power solution is currently under development, which would further extend deployment capability to berths without accessible electrical infrastructure. The private 4G network node (eNodeB) was purposely built on a mobile cart, allowing rapid repositioning and redeployment at different berths or port facilities without requiring permanent network infrastructure modifications.

Camera placement relied on fixed elevated structures along the quay, such as lighting posts, which are standard features of most commercial port facilities worldwide. Different berth layouts can, therefore, be accommodated by repositioning the camera devices on available elevated structures, provided they offer a clear line of sight to the vessel hull. Similarly, the bollard-based metric calibration strategy is not restricted to bollards specifically, and any fixed structure of known height with a well-defined geometric relationship to the vessel hull can serve as a reference object, making the calibration approach adaptable to different quay configurations.

Regarding vessel types, the current framework and trained segmentation models are optimized for bulk carriers, which represent the primary operational focus of this work. Extension to other vessel classes, such as container ships or tankers, would require retraining the YOLOv8 segmentation models with representative imagery from those vessel types, given differences in hull geometry, freeboard height, and visual appearance. The modular nature of the framework, in which segmentation models are independently trained per viewpoint and vessel type, facilitates such extensions without requiring architectural modifications to the measurement pipeline or communication system.

Taken together, these characteristics suggest that the proposed framework can, in principle, be deployed across a broad range of bulk carrier operations at different port facilities, subject to site-specific camera placement and model training requirements. It is also a deliberate and meaningful step toward automation of draft-survey operations, rather than a complete replacement of existing procedures. The relationship between the estimated hull height (*H*) and the vessel draft is straightforward: as cargo is progressively loaded and the vessel settles deeper in the water, the emergent hull height decreases proportionally, which is a consistent trend observed throughout the experimental results in Section 5. This geometric relationship forms the foundation upon which a fully automated draft reading system can be built.

It is acknowledged in measurement science and engineering that complex automated systems are rarely achieved in a single development step. Progress toward full automation requires first establishing a reliable data acquisition infrastructure, then validating the geometric measurement pipeline, and subsequently integrating the hydrostatic models and density corrections necessary for statutory draft-survey accuracy. This work addresses the first stage, demonstrating that continuous, non-intrusive, and reliable hull geometry data can be acquired and processed in a real operational port environment without any human intervention onboard or surrounding the vessel. Without this foundational capability, no further automation would be possible.

### Limitations: Hardware, Operational and Regulatory

Despite the promising results demonstrated in this work, several limitations must be acknowledged, spanning both technical and operational dimensions.

The private 4G network infrastructure deployed in this work is based on the eNodeB, which operates in Time Division Duplexing (TDD) mode under standard LTE TDD bands. In Dual Carrier (DC) mode, the system supports a maximum of up to 96 + 96 simultaneously connected devices, with peak downlink and uplink data rates of 220 Mbps and 28 Mbps, respectively, under a 2 × 20 MHz bandwidth configuration. While these specifications are more than sufficient for the two-camera deployment presented in this work, scaling the system to a larger number of simultaneous camera devices, for instance, to monitor multiple vessels or multiple berths simultaneously, would require careful network planning to ensure that bandwidth and user capacity limits are not exceeded. Furthermore, the system’s latency of 30 ms, while acceptable for the real-time monitoring application described here, may require further optimization for applications demanding a higher temporal resolution or tighter synchronization between devices.

The edge devices are constrained by their available computational resources, limiting on-device processing to image capture, buffering, and transmission. All segmentation and geometric measurement processing is performed on the Port Server, meaning that any failure or unavailability of the Port Server directly impacts the system’s ability to produce hull-height estimates in real time. Although the Remote Application provides failover processing capability, the transition between primary and backup processing nodes has not been formally characterized in terms of switchover time or data continuity.

Nighttime operability represents a further hardware limitation. As observed in the experimental results, the segmentation models produce no valid hull detections during nighttime hours when ambient illumination is insufficient. This constrains the system’s reliability during night-shift operation, which may limit its applicability for continuous 24 h monitoring. Future iterations of the system will address this through the integration of dedicated infrared cameras or low-light imaging sensors.

Beyond the technical constraints, the deployment of the proposed system in real port environments introduces a series of operational and regulatory challenges that must be carefully managed.

Port facilities are highly regulated environments governed by strict safety, security, and access control policies. The installation of camera systems and wireless network infrastructure requires formal authorization from port authorities, which may involve lengthy approval processes and compliance with local regulations regarding data privacy, radio-frequency emissions, and physical equipment placement. In the case of the Port of Santos, the experiments were conducted in close cooperation with the Santos Port Authority, whose institutional support was essential for obtaining the necessary access permissions and facilitating equipment installation at elevated positions along the quay.

The placement of cameras was constrained by the physical layout of port infrastructure, including the presence of cranes, conveyors, ship loaders, and other terminal equipment that may obstruct the camera’s line of sight to the vessel hull. As observed during the experimental campaign, partial occlusions of the hull by port equipment occurred periodically, affecting the continuity of hull-height measurements during those intervals.

The georeferenced bollard used as the metric calibration reference in this work is a feature specific to the Port of Santos infrastructure. While similar certified reference structures are expected to be available at other major port facilities, their availability, accessibility, and dimensional documentation cannot be guaranteed across all deployment sites, and site-specific calibration procedures may be required.

Finally, the current framework has been validated exclusively on bulk carrier vessels at a single port facility. Generalizing the system to other vessel types, such as container ships, tankers, or RO-RO vessels, would require improved model training with representative imagery, and potentially adapting the geometric measurement pipeline to account for differences in hull geometry and freeboard characteristics. Similarly, validation across different port environments, sea states, and climatic conditions remains an important direction for future work.

## 7. Conclusions

This study presented and validated a computer vision framework for automated draft-survey support based on hull-height estimation, deployed at the Port of Santos, Brazil, in cooperation with the Santos Port Authority. The proposed system combines fixed quay-side cameras, a mobile private 4G network architecture, customized YOLOv8 instance-segmentation models, and a local physical reference for pixel-to-metric calibration, enabling continuous and non-intrusive monitoring of vessel loading state without relying on draft mark detection, digit recognition, or explicit waterline identification. By avoiding these uncertainty-prone steps, the framework addresses persistent limitations present in both traditional and existing automated draft-survey approaches, representing a methodological shift toward geometry-based continuous hull monitoring.

Field experiments monitoring a Panamax bulk carrier over approximately 6.5 days processed more than 34,000 images per camera at an average rate of 3.7 images per min. Bow and stern segmentation models achieved mAP50-95 mask scores of 0.980 and 0.965, respectively, confirming precise and stable hull boundary delineation under real operational conditions including variable lighting, background clutter, and partial occlusions. Hull height decreased consistently from 8.27 m to 4.64 m at the bow and from 7.98 m to 3.98 m at the stern over the loading period, with coherent temporal trends across independent viewpoints, confirming that the measured variation reflects physical loading behavior rather than measurement noise. The private 4G communication architecture maintained stable image transmission throughout the campaign, with an internal buffering mechanism ensuring data continuity during transient network disturbances.

While the present framework does not yet directly compute cargo mass, which would require full hydrostatic data and density corrections, it establishes a reliable geometric basis for continuous monitoring of vessel loading dynamics, relative trim assessment, and event-based timeline reconstruction, complementing rather than replacing conventional draft surveys at this stage. Future work will focus on integrating hydrostatic models for cargo mass estimation, extending nighttime operability through enhanced illumination or low-light imaging solutions, validating the approach across a broader range of vessel types and port environments, and investigating the use of 5G networks to further improve transmission reliability and latency.

## Figures and Tables

**Table 1:** Key hardware specifications of the deployed equipment.

Specification	Raspberry Pi 4 Model B	Raspberry Pi Camera Module 3	Elsys Antenna
Core	Broadcom BCM2711, Quad-core Cortex-A72 (ARMv8) 64-bit SoC @ 1.8 GHz; 8 GB LPDDR4-3200 SDRAM	Sony IMX708 sensor	–
Image capture	–	11.9 MP still resolution; Sensor resolution: 4608 × 2592 px; Pixel size: 1.4 µm × 1.4 µm; Optical size: 1/2.43″	–
Optics	–	Focal length: 4.74 mm; Focal ratio: f/1.8; HFoV: 66°; VFoV: 41°; Motorised focus; Depth of field: ∼10 cm to ∞	–
Connectivity	Gigabit Ethernet; IEEE 802.11ac (2.4/5.0 GHz); Bluetooth 5.0/BLE; 40-pin GPIO; 2× USB 3.0; 2× USB 2.0	2-lane MIPI CSI port	RJ45 Ethernet (IEEE 802.3, 10/100 Mbps); PoE support; Nano SIM slot
Network performance	–	–	LTE DL: 70 Mbps max; LTE UL: 50 Mbps max; HSPA DL: 21 Mbps max; MIMO 2 × 2 (B4/B66)
Frequency bands	–	–	4G: 600 (B71), 700 (B12, B13, B14), 850 (B5), 1700 (B4, B66), 1900 (B2) MHz
Antenna gain	–	–	6 dBi 700–960 MHz; 7.9 dBi 1710–1910 MHz; 10 dBi @ 1920–2700 MHz
Power supply	5 V DC via USB-C or GPIO	Powered via Raspberry Pi	12–24 V DC
Storage	Micro-SD card	–	–

**Table 2:** Total of images used to train the models.

	Bow	Stern
Labeled images	608	532
Training	427	425
Validation	122	68
Testing	59	39

**Table 3:** Data augmentation parameters used for training the YOLOv8 hull-segmentation models.

Parameter	Default	Description
hsv_h	0.015	Random variation in hue in the HSV color space to simulate changes in ambient lighting and color temperature caused by sunlight and atmospheric conditions.
hsv_s	0.7	Random adjustment of saturation to account for variations in hull paint appearance, wet surfaces, reflections, and camera exposure.
hsv_v	0.4	Random adjustment of brightness (value channel) to improve robustness under different illumination levels, including overcast skies and strong sunlight.
degrees	5	Small random rotations ($\pm 5^{\circ }$) to model minor camera misalignment and vessel orientation variability during mooring.
translate	0.05	Random horizontal and vertical translations (up to 5% of image size) to simulate small shifts in camera position and framing.
scale	0.2	Random scaling of the image (±20%) to account for variations in vessel distance and camera zoom while preserving hull geometry.
shear	2	Shearing transformation ($\pm 2^{\circ }$) to increase robustness to slight perspective distortions caused by oblique viewing angles.
perspective	0.0005	Minor perspective warping to model non-planar projection effects without introducing unrealistic hull deformation.
flipud	0	Vertical flipping disabled, as upside-down hull appearances are physically implausible in maritime environments.
fliplr	0.5	Horizontal flipping applied with 50% probability to exploit hull symmetry and improve generalization across left- and right-view cameras.
mosaic	1	Mosaic augmentation enabled to combine multiple images into a single training sample, increasing background diversity and robustness to occlusions.
mixup	0.1	MixUp augmentation with 10% probability to reduce overfitting by blending image–label pairs and smoothing decision boundaries.

**Table 4:** Summary of image acquisition and transmission statistics for devices 102 and 106.

Device	Images	Start Time	End Time	Continuous Time	Rate (Images/min)
102	34,754	21 November 2025 10:50:17	27 November 2025 23:59:45	6 days 13:09:12 (565,752 s)	3.68
106	35,035	21 November 2025 10:50:18	27 November 2025 23:59:59	6 days 13:09:41 (565,781 s)	3.71

**Table 5:** Kalliopi L (IMO 9233284) port call timeline of events.

No.	Process	Observed Timestamp	Statement of Facts Timestamp
1	Berth request	–	–
2	Arrival at anchorage	–	9 November 2025 10:30:00
3	Agreement with operator	–	–
4	NOR	–	9 November 2025 10:48:00
5	Programming of ships	–	–
6	Canal approach	–	21 November 2025 20:18:00
7	Tug and pilotage request	–	21 November 2025 22:36:00
8	Inbound canal navigation	–	21 November 2025 23:48:00
9	Berthing maneuver	21 November 2025 23:06:53	–
10	Berthing	22 November 2025 00:05:58	22 November 2025 00:12:00
11	Pre-operational procedures	22 November 2025 00:12:42	22 November 2025 00:50:00
12	Ship operation	22 November 2025 01:54:57	22 November 2025 02:00:00
13	Post-operational procedures	26 November 2025 16:04:15	26 November 2025 16:00:00
14	Unberthing schedule	26 November 2025 21:55:24	26 November 2025 18:00:00
15	Maneuver scheduling	–	–
16	Tug and pilotage services	26 November 2025 22:13:06	–
17	Unberthing maneuver	26 November 2025 22:25:23	–
18	Departure maneuver	26 November 2025 22:32:00	–
19	Outbound canal navigation	–	–
20	Exit the canal	–	–

## Data Availability

Data are contained within the article.
